# Differential Treatment Effects for Renal Transplant Recipients With DSA-Positive or DSA-Negative Antibody-Mediated Rejection

**DOI:** 10.3389/fmed.2022.816555

**Published:** 2022-01-31

**Authors:** Marius Andreas Koslik, Justa Friebus-Kardash, Falko Markus Heinemann, Andreas Kribben, Jan Hinrich Bräsen, Ute Eisenberger

**Affiliations:** ^1^Department of Nephrology, University Hospital Essen, University of Duisburg-Essen, Essen, Germany; ^2^Institute for Transfusion Medicine, Transplantation Diagnostics, University Hospital Essen, University of Duisburg-Essen, Essen, Germany; ^3^Nephropathology Unit, Hannover Medical School, Institute of Pathology, Hanover, Germany

**Keywords:** antibody-mediated rejection, donor-specific antibody, treatment, IVIG (intravenous immunoglobulin) administration, plasmapheresis, maintenance immunosuppression

## Abstract

**Background:**

Antibody-mediated rejection (ABMR) is the main cause of renal allograft loss. The most common treatment strategy is based on plasmapheresis plus the subsequent administration of intravenous immunoglobulin (IVIG). Unfortunately, no approved long-term therapy is available for ABMR. The current study was designed to analyze the effect of various ABMR treatment approaches on allograft survival and to compare treatment effects in the presence or absence of donor-specific antibodies (DSAs).

**Methods:**

This single-center study retrospectively analyzed 102 renal allograft recipients who had biopsy-proven ABMR after transplant. DSA was detectable in 61 of the 102 patients. Initial standard treatment of ABMR consisted of plasmapheresis (PS) or immunoadsorption (IA), followed by a single course of IVIG. In case of nonresponse or recurrence, additional immunosuppressive medications, such as rituximab, bortezomib, thymoglobulin, or eculizumab, were administered. In a second step, persistent ABMR was treated with increased maintenance immunosuppression, long-term therapy with IVIG (more than 1 year), or both.

**Results:**

Overall graft survival among transplant patients with ABMR was <50% after 3 years of follow-up. Compared to the use of PS/IA and IVIG alone, the use of additional immunosuppressive medications had no beneficial effect on allograft survival (*p* = 0.83). Remarkably, allografts survival rates were comparable between patients treated with the combination of PS/IA and IVIG and those treated with a single administration of IVIG (*p* = 0.18). Renal transplant patients with ABMR but without DSAs benefited more from increased maintenance immunosuppression than did DSA-positive patients with ABMR (*p* = 0.01). Recipients with DSA-positive ABMR exhibited significantly better allograft survival after long-term application of IVIG for more than 1 year than did recipients with DSA-negative ABMR (*p* = 0.02).

**Conclusions:**

The results of our single-center cohort study involving kidney transplant recipients with ABMR suggest that long-term application of IVIG is more favorable for DSA-positive recipients, whereas intensification of maintenance immunosuppression is more effective for recipients with DSA-negative ABMR.

## Introduction

Despite all efforts, long-term renal allograft survival is limited to an average of 11 to 15 years (1). The cause of allograft failure is multifactorial. However, antibody-mediated rejection (ABMR) is the main factor contributing to progressive deterioration of allograft function and subsequent allograft loss (1).

Pathophysiological knowledge of the ABMR process has been increasing in recent years. One of the key elements for the diagnosis of ABMR is the formation of donor-specific antibodies (DSAs) (2). DSAs directed against mismatched human leukocyte antigens (HLA) class I and II attach to the endothelium, triggering complement activation via the classic pathway and inducing Fc gamma receptor–dependent effects on the activation of natural killer cells and macrophages. Membrane attack complex (MAC) activated by C1q is responsible for inflammation in the vascular endothelium, generating direct irreversible injury of the allograft (3). Histologic features, such as glomerulitis and peritubular capillaritis, as well as chronic glomerulopathy, indicate endothelial damage. In addition, microvascular injury stimulates platelet activation, resulting in the development of microthrombi (4, 5). C4d is a specific correlate of complement cascade activation initiated by DSAs. As a degradation product of C4, C4d binds to endothelium (3); often rendering C4d deposits detectable in biopsy samples from allografts in patients with ABMR (6). Thus, C4d deposition in renal allografts is one diagnostic criterion for acute and chronic ABMR (7).

Preformed DSAs are present in one third of recipients in whom early acute ABMR takes a severe course (5, 8). However, *de novo* DSAs can develop in nonsensitized patients after transplant. Although ABMR is more common in the late posttransplant course, early ABMR can occur within the first 6 months after transplant (3, 9, 10). ABMR appears in the context of under immunosuppression and is related to the production of DSAs (9, 10).

However, several reports have documented the histologic picture of ABMR in the absence of detectable DSAs (11–17). Circulating DSAs in the presence of ABMR-compatible histologic lesions were absent in as many as 27% of patients with ABMR (12). In addition to the possibility that current techniques cannot detect some anti–HLA-DSA antibodies, DSA-negative ABMR cases may be explained by the occurrence of non-HLA antibodies after renal transplant, the presence of HLA-specific memory B cells, and intragraft deposition of DSAs (14–17). Although published information about DSA-negative ABMR is still limited, the comparison of allograft outcomes between DSA-negative ABMR and DSA-positive ABMR has yielded controversial results (11–13).

Therapy for ABMR is one of the main challenges facing transplant medicine. Currently, no approved treatments for chronic ABMR exist (1, 5). Plasmapheresis (PS) in combination with high-dose intravenous immunoglobulin (IVIG) has proved to be effective in several trials and is the current standard of care for acute ABMR (1, 5). However, the quality of the evidence supporting this treatment regimen is low (1, 18). Therefore, there is an unmet need for new, innovative therapeutic approaches. Recent studies compared the ability of three potential ABMR therapies (the B cell–depleting antibody rituximab; an inhibitor of proteasome, bortezomib, which interferes with alloantibody-producing plasma cells; and an antibody targeting a terminal component of complement pathway, eculizumab) to prevent the genesis of MAC (1, 3, 5). Unfortunately, the results of these therapeutic strategies were disappointing (1, 3, 5). The quality of the existing data is also moderate because most of the studies were small pilot studies and were consequently underpowered (1, 5). Additionally, a proper comparison of previous studies is difficult because intervention protocols vary strongly between centers and because the studies did not account for the various heterogeneous phenotypes of ABMR that have been established in recent years by the evolution of the Banff classification of ABMR (1, 19).

The study reported here analyzed the effect of a stepwise treatment approach for ABMR on allograft survival and compared treatment effects in the presence or absence of DSAs. Additionally, we estimated the importance of various factors associated with ABMR for allograft loss.

## Materials and Methods

### Study Population

**Figure 1:**
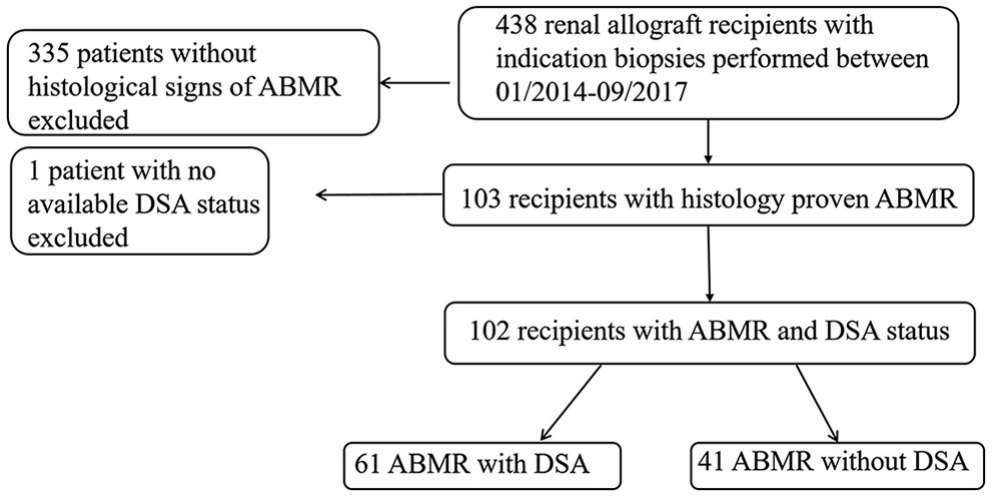
Study population flow chart. ABMR, antibody-mediated rejection; DSA, donor-specific antibody.

Between January 2014 and September 2017, 438 adult recipients of renal allografts, most of whom were treated at the University Hospital Essen, underwent a total of 833 renal transplant biopsies. All biopsies were performed for cause, and samples were analyzed according to the latest available Banff grading criteria (19, 20). Several patients underwent more than one renal biopsy during the study period (on average, 2 renal biopsies were performed per patient). In such cases we considered the earliest and most representative biopsy. No protocol biopsies were performed during the study period. As shown in Figure 1, biopsy-proven ABMR was detected in 103 recipients. For one patient no report on DSAs was available, and this patient was excluded from further analysis. Therefore, 102 recipients with ABMR and available DSA status were included in the current study. Control biopsies performed after the completion of standard treatment of ABMR were used to evaluate the effectiveness of previous treatment. Experienced nephropathologists examined all renal transplant specimens with light microscopy and immunohistochemical analyses. The retrospective single-center study was approved by the institutional ethics board (19-8097-BO).

Clinical and laboratory data were collected by a review of the electronic medical record. Allograft survival was followed up for at least 3 years after the performance of the first biopsy that showed evidence of ABMR. Clinical data on all therapy approaches that were used to cure ABMR were collected for as long as 3 years after the initial biopsy.

### Treatment Approaches for ABMR

In our stepwise treatment approach, the first step of initial standard therapy for acute ABMR involved 3–5 runs of PS, immunoadsorption (IA), or both, with subsequent intravenous administration of IVIG at a dosage of 0.5–0.8 g/kg over 3 days (Table 1). If there was no response to initial standard therapy, or if ABMR was detected by a follow-up biopsy, additional immunosuppressive drugs such as rituximab, bortezomib, eculizumab, or thymoglobulin were administered off-label (Table 1). The second step, for treatment of biopsy-proven persistent ABMR, involved an increase in maintenance immunosuppression, long-term therapy with IVIG for more than 1 year, or both (Table 1).

**Table 1:** Overview of therapies used to treat biopsy-proven antibody-mediated rejection among 102 renal allograft recipients.

**Primary therapy of ABMR**	**All patients**	**Patients with DSA^**+**^ ABMR**	**Patients with DSA^**−**^ABMR**	***p*-value**
**No therapy**	1	0	1	n.c.
**Thymoglobulin alone**	1	1	0	n.c.
**Eculizumab alone**	1	1	0	n.c.
**Intensification of maintenance immunosuppression** **+** **eculizumab**	2	0	2	n.c.
**IVIG alone**	13	6	7	0.285
**IVIG** **+** **IA/PS**	84	53	31	0.145
*IVIG* + *PS*	*75*	*46*	*29*	*n.c*.
*IVIG* + *IA*	*9*	*7*	*2*	*n.c*.
*IVIG* + *IA/PS without add-on therapy*	*46*	*27*	*19*	*0.837*
*IVIG* + *IA/PS* + *add-on therapy*	*38*	*26*	*12*	*0.174*
*IVIG* + *IA*/*PS* + *rituximab*	*10*	*8*	*2*	*n.c*.
*IVIG* + *IA/PS* + *bortezomib*	*11*	*8*	*3*	*n.c*.
*IVIG* + *IA/PS* + *thymoglobulin*	*9*	*5*	*4*	*n.c*.
*IVIG* + *IA/PS* + *eculizumab*	*3*	*1*	*2*	*n.c*.
*IVIG* + *IA/PS* + *multiple add-on therapies*	*5*	*4*	*1*	*n.c*.
**Secondary therapy of ABMR**				
**Long-term IVIG**	**36**	**27**	**9**	**0.021**
Long-term IVIG alone	6	5	1	0.228
Long-term IVIG + IA/PS without add-on therapy	11	8	3	0.357
Long-term IVIG + IA/PS with add-on therapy	19	14	5	0.173

*ABMR, antibody-mediated rejection; DSA, donor-specific antibody; IA, immunoadsorption; IVIG, intravenous immune globulin; n.c., not calculated; PS, plasmapheresis*.

*Bold values are significant values (p < 0.05)*.

### Immunological Analyses

Pretransplant lymphocytotoxic crossmatches were negative for all recipients. For HLA typing of recipients and donors, DNA was isolated from peripheral blood samples with spin columns (Qiagen, Hilden, Germany) or with an automated system using magnetic separation technology (Chemagic, Chemagen PerkinElmer, Baesweiler, Germany). HLA-class I (HLA-A, -B, -C) and II (HLA-DRB1, -DQB1) typing was performed at the first field resolution level with sequence-specific primers (polymerase chain reaction sequence-specific primer method) or alternatively with sequence-specific oligonucleotides (LABType SSO method, both provided by One Lambda/Thermo Fisher Inc., Canoga Park, CA, USA) (21).

All patients were screened for anti-HLA antibodies before transplant. Anti-HLA antibody status after transplant was monitored at months 3, 6, and 12 after transplant and annually thereafter. Additional screening was performed if allograft dysfunction occurred. Pretransplant sensitization status was determined with the standard immunoglobulin G (IgG) complement-dependent cytotoxicity test in combination with a Luminex-based LABScreen Mixed beads assay (One Lambda, Thermo Fisher Scientific, Inc.), which was used to identify the antibodies against HLA classes I and II. If the results of the LABScreen Mixed beads assay were positive, anti-HLA antibodies were further characterized in terms of IgG alloantibody specificity for HLA-A, -B, -C, -DR, -DP, and -DQ with LABScreen single-antigen bead (SAB) assays (One Lambda, Thermo Fisher Scientific Inc.) according to the manufacturer's protocols. All beads with normalized median fluorescence intensity (MFI) values higher than 1.000 were considered to be positive for anti-HLA antibodies. To address a potential effect of interfering antibodies or prozone effects on our MFI analyses, all patient sera included in this study have been treated with ethylenediaminetetraacetic acid (EDTA) prior to Luminex-based assay testing (22).

### Statistical Analyses

Categorical variables were expressed as numbers and percentages. Comparisons between groups were made with the χ^2^ test for categorical variables and with the Mann–Whitney test for continuous variables. Allograft survival among patients with ABMR was illustrated with Kaplan–Meier survival curves and analyzed with the log-rank test. To assess independent factors influencing renal allograft survival, we performed a multivariable Cox regression analysis and calculated the hazard ratio (HR) and the 95% confidence interval (CI). Variables for multivariate analysis were selected based on the results of univariate analysis. For all tests, statistical significance was set at the level of *p* ≤ 0.05. All data analyses were performed with GraphPad Prism version 6 (GraphPad Software, Inc., La Jolla, CA, USA) and IBM SPSS Statistics version 23 (IBM Corp., Armonk, NY, USA).

## Results

### Patient Characteristics

Of the 438 renal allograft recipients who underwent allograft biopsy between January 2014 and September 2017, 102 were found to have biopsy-proven ABMR with known DSA status, one patient with ABMR was excluded due to missing DSA status. The remaining 335 renal allograft recipients who received a primary biopsy for cause showed the following results: Borderline rejection (Banff category 3) in 60 and T-cell mediated rejection (Banff category) in 46, Banff category 6 in 201 (with CNI-toxicity in 51, polyoma virus nephropathy in 12, acute reversible tubular necrosis in 94, signs of infection in 10, recurrence of primary renal disease in 34), Banff category 5 in 1, normal renal tissue in 4, non-representative renal biopsy in 23.

Clinical and demographic characteristics of these 102 patients with histologic features of ABMR are summarized in Table 2. Of these patients, 24 (24%) underwent renal allograft biopsy within the first year after transplant. At the time of biopsy, 101 of the 102 patients were receiving maintenance immunosuppression including steroids; 76 patients (75%) were treated with tacrolimus as a calcineurin inhibitor, 16 (16%) with cyclosporin A. Most patients (80; 78%) were receiving maintenance immunosuppression with mycophenolic acid (MMF) or mycophenolate mofetil (MPA) and 12 (12%) with everolimus. DSAs were detected in 61 (60%) patients with histologic evidence of ABMR. The remaining 41 patients with ABMR had a negative DSA status at the time of diagnosis. Among DSA-positive patients, 14 (14%) exhibited anti–HLA-DSA class I antibodies and 35 (34%) exhibited DSA class II antibodies. Twelve (12%) recipients exhibited anti–HLA-DSA class I and class II antibodies simultaneously.

**Table 2:** Baseline characteristics of 102 renal allograft recipients with biopsy-proven antibody-mediated rejection.

**Variables**	**All patients**	**Patients with DSA^**+**^ ABMR**	**Patients with DSA^**−**^ABMR**	***p*-value**
	***n =* 102**	***n =* 61**	***n =* 41**	
**Characteristics at the time of Tx/biopsy**				
Number of men, *n* (%)	47 (46.1)	31 (50.8)	16 (39.0)	0.241
Recipient age, median (IQR)	**43 (26,75–55.25)**	**37 (21.5–52.0)**	**49 (39.0–62.0)**	**<0.001**
Recipient age at the time of biopsy, median (IQR)	**50.5 (32.25–60.25)**	**47 (27.0–56.5)**	**54 (43.5–65.0)**	**0.008**
Time between Tx and biopsy in days, median (IQR)	**1,550 (451–3,605)**	**2,720 (1,151–4,142)**	**705 (34–1,741)**	**<0.001**
Allograft biopsy <1 year after Tx, *n* (%)	**24 (23.5)**	**5 (8.2)**	**19 (46.3)**	**<0.001**
Allograft biopsy >1 year after Tx, *n* (%)	**78 (76.5)**	**56 (91.8)**	**22 (53.7)**	**<0.001**
Living donor, *n* (%)	26 (25.5)	19 (31.1)	7 (17.1)	0.081
Cold ischemia time (h:min), median (IQR)	12:21 (5:27–17:09)	11:02 (2:36–17:00)	13:35 (7:36–17:31)	0.198
Previous transplants, *n* (%)	**20 (19.6)**	**7 (11.5)**	**13 (31.7)**	**0.019**
HLA class I and II mismatch (HLA-A, -B, -DR), median (IQR)	**3 (2–4)**	**3 (2–4)**	**2 (0–3)**	**0.016**
HLA class I mismatch (HLA-A, -B), median (IQR)	**2 (1–2)**	**2 (1–3)**	**1 (0–2)**	**0.020**
HLA class II mismatch (HLA-DR), median (IQR)	1 (0–1)	1 (0–2)	1 (0–1)	0.050
ABO-incompatible Tx, *n* (%)	0 (0)	0 (0)	0 (0)	–
Current PRA ≥ 5%, *n* (%)	22 (21.6)	13 (21.3)	9 (22.0)	0.939
Current PRA ≥ 20%, *n* (%)	13 (12.7)	7 (11.5)	6 (14.7)	0.639
Anti-HLA–DSA, *n* (%)	61 (59.8)	61 (100.0)	–	–
Anti-HLA–DSA class I, *n* (%)	14 (13.7)	14 (23.0)	–	–
Anti-HLA–DSA class II, *n* (%)	35 (34.3)	35 (57.4)	–	–
Anti-HLA–DSA class I and II, *n* (%)	12 (11.8)	12 (19.7)	–	–
Peak MFI of DSA, median (IQR)	8,500 (3,150–17,650)	8,500 (3,150–17,650)	–	–
Sum of MFI, median (IQR)	9,800 (3,300–21,650)	9,800 (3,300–21,650)	–	–
**Immunosuppression at the time of biopsy**				
Steroids, *n* (%)	101 (99)	61 (100)	40 (97.6)	0.402
Cyclosporine A, *n* (%)	16 (15.7)	12 (19.7)	4 (9.8)	0.177
Tacrolimus, *n* (%)	76 (74.5)	47 (77.0)	29 (70.7)	0.473
MMF or MPA, *n* (%)	80 (78.4)	45 (73.8)	35 (85.4)	0.163
Belatacept, *n* (%)	**4 (3.9)**	**0 (0)**	**4 (9.8)**	**0.024**
Everolimus, *n* (%)	**12 (11.8)**	**3 (4.9)**	**9 (22.0)**	**0.012**
Sirolimus, *n* (%)	2 (2.0)	1 (1.6)	1 (2.4)	1.000
Azathioprine, *n* (%)	2 (2.0)	1 (1.6)	1 (2.4)	1.000

*ABMR, antibody-mediated rejection; anti-HLA, anti–human leukocyte antigen; DSA, donor-specific antibody; IQR, interquartile range; MFI, mean fluorescence intensity; MMF, mycophenolate mofetil; MPA, mycophenolic acid; PRA, panel-reactive antibodies, Tx, transplantation*.

*Bold values are significant values (p < 0.05)*.

Comparing patients with DSA-positive ABMR and patients with DSA-negative ABMR, we noted that a larger proportion of patients in the DSA-negative group had undergone previous transplants (Table 2). Recipients without DSA were older and were more likely to have undergone a renal biopsy during the first year after transplant at first evidence of ABMR (Table 2). Mismatches in HLA class I and class II were more frequent in DSA-positive patients with ABMR than in ABMR patients without DSA finding (Table 2). Immunosuppressive regimens at the time of biopsy were mainly comparable except that the use of everolimus and belatacept was more common among DSA-negative patients (Table 2).

Overall allograft survival among all kidney transplant patients with ABMR was <50% at the 3-year follow-up.

### Late ABMR and Positive C4d Status Were the Main Risk Factors for Allograft Failure

First, we analyzed the effect of several variables associated with ABMR on allograft survival among the 102 recipients with biopsy-proven ABMR. We selected several relevant factors, such as DSA status, C4d positivity, occurrence of ABMR within the first year after transplant, and histologic signs of various Banff categories in addition to ABMR so that we could evaluate the potential effect of these variables on allograft outcome.

**Figure 2:**
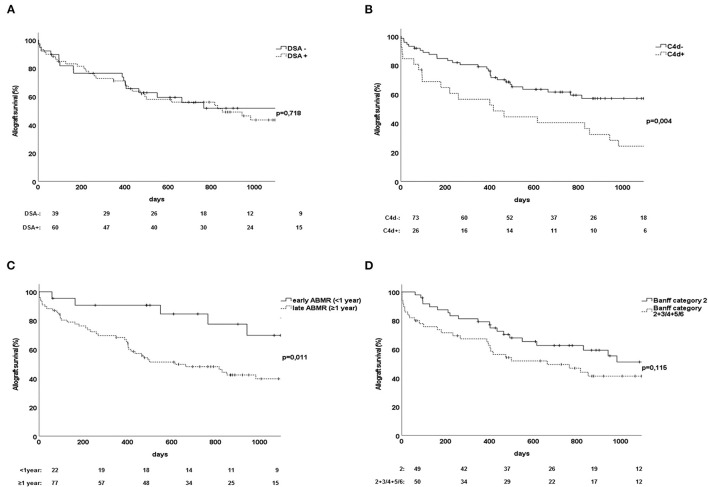
Association of various factors related to accelerated allograft loss due to ABMR among 102 transplant recipients. **(A)** Influence of DSA status of ABMR on renal allograft survival. **(B)** Influence on renal allograft survival of C4d deposits among patients with biopsy-proven ABMR. **(C)** Allograft survival in relation to ABMR detected within the first year after transplant. **(D)** Allograft survival among patients with ABMR and Banff category 2 only compared to Banff category 2 in combination with other pathologic lesions (Banff category 3 or 4 and/or 5 or 6). ABMR, antibody-mediated rejection; DSA, donor-specific antibody.

Renal allograft survival rates were comparable between recipients with DSA-positive or DSA-negative ABMR, a finding suggesting that DSA status had no significant effect on allograft loss in the present cohort (*p* = 0.72, Figure 2A).

Renal allograft biopsies found evidence of C4d deposition among 26 patients in our cohort. C4d positivity was associated with significantly worse allograft survival at the follow-up 3 years after transplant (*p* = 0.004, Figure 2B).

As expected, the occurrence of biopsy-proven ABMR within the first year after transplant, referred as early ABMR, reflected an advantage in terms of allograft survival compared to late ABMR (*p* = 0.01, Figure 2C). However, many recipients with early ABMR exhibited no DSAs (Table 2).

Besides humoral rejection within Banff category 2, a proportion of patients showed additional histologic signs related to other Banff categories. There were no significant differences in allograft survival rates between patients with ABMR alone and those with co-occurrence of ABMR and T cell–mediated rejection or borderline changes, although we observed a slight trend toward earlier occurrence of allograft loss among patients with characteristics of other Banff categories in addition to Banff category 2 (Figure 2D).

Univariate and subsequent multivariate analyses found that late ABMR and C4d were independent risk factors for allograft failure among patients with ABMR after renal transplant (Table 3).

**Table 3:** Results of univariate and multivariate analyses identifying risk factors and protective factors for allograft failure among 102 renal allograft recipients with ABMR.

	**HR**	**CI (95%)**	***p*-value**
	***n =* 102**		
**Univariate analysis**			
**Risk factors**			
DSA^+^	1.116	0.615–2.023	0.719
Previous transplants	0.986	0.462–2.134	0.986
C4d^+^	**2.285**	**1.275–4.098**	**0.006**
ABMR ≥ 1 year	**3.116**	**1.231–7.887**	**0.016**
Banff category 2	0.681	0.383–1.211	0.191
Banff category 2 + 3/4	0.922	0.467–1.820	0.816
Banff category 2 + 5/6	1.445	0.715–2.921	0.305
Banff category 2 + 3/4 + 5/6	2.238	0.881–5.685	0.090
Recipient age at the time of biopsy ≥ 50 years	0.986	0.555–1.751	0.962
Acute ABMR	0.914	0.519–1.612	0.757
Acute+chronic-active ABMR	1.332	0.712–2.490	0.369
Chronic-active ABMR	0.837	0.426–1.645	0.606
**Therapy regimen**			
Add-on therapies	0.937	0.525–1.672	0.825
Intensification of maintenance immunosuppression	**0.372**	**0.167–0.831**	**0.016**
Long-term IVIG	0.637	0.348–1.169	0.145
**Multivariate analysis**			
C4d^+^	**2.522**	**1.405–4.526**	**0.002**
ABMR ≥ 1 year	**2.604**	**0.982–6.901**	**0.054**
Intensification of maintenance immunosuppression	0.470	0.202–1.091	0.079

*ABMR, antibody-mediated rejection; CI, confidence interval; DSA, donor-specific antibody; HR, hazard ratio; IVIG, intravenous immune globulin*.

*Bold values are significant values (p < 0.05)*.

### Administration of IVIG Alone Was as Effective as Administration of the Combination of PS/IA With IVIG for the Treatment of ABMR

**Figure 3:**
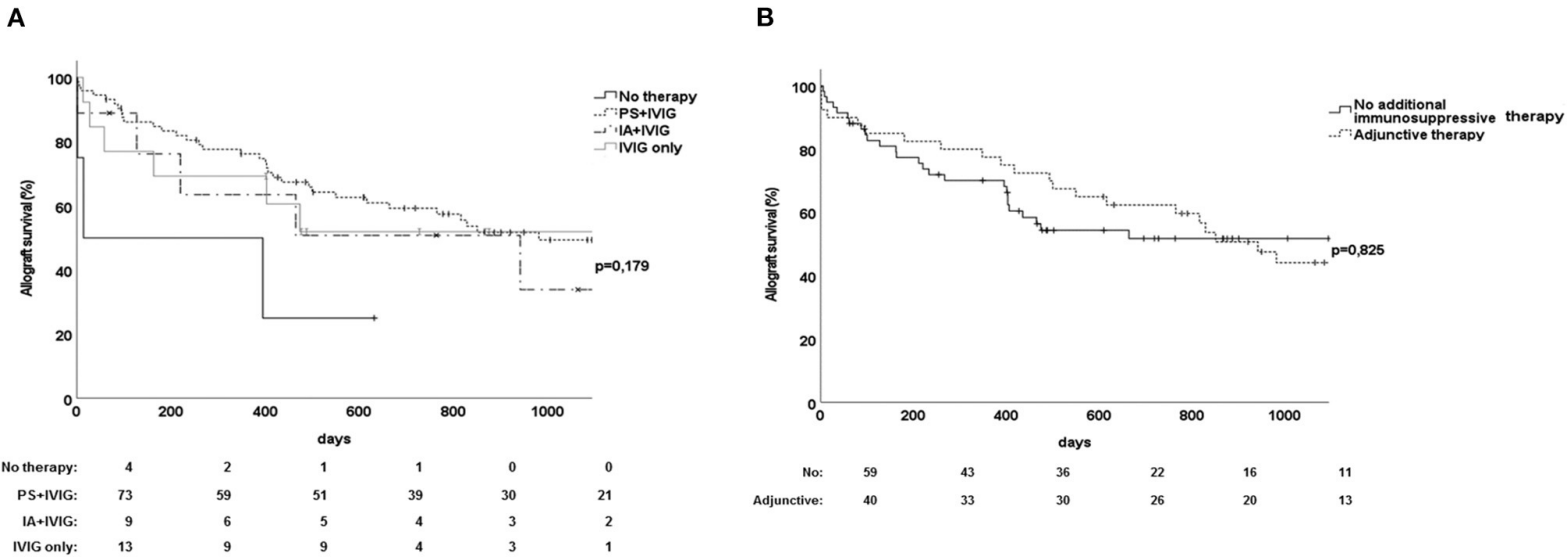
Effect of various treatment approaches on renal allograft survival among 102 recipients with antibody-mediated rejection. **(A)** Comparison between the use of plasmapheresis plus IVIG vs. immunoadsorption with IVIG and IVIG alone. **(B)** Effect on renal allograft survival of the administration of adjunctive immunosuppressive drugs in addition to standard therapy among recipients with ABMR. Adjunctive immunosuppressive drugs were rituximab (*n* = 10), bortezomib (*n* = 11), thymoglobulin (*n* = 9), or eculizumab (*n* = 3). ABMR, antibody-mediated rejection; IA, immunoadsorption; IVIG, intravenous immune globulin; PS, plasmapheresis.

Among 102 renal allograft recipients with evidence of ABMR, 84 patients were initially treated with PS, IA, or both and subsequent application of IVIG. As shown in Figure 3A, we observed no difference in renal allograft survival rates between ABMR treatment with plasmapheresis or immunoadsorption (*p* = 0.44). The remaining 13 recipients (Table 1) were treated with IVIG alone without any application of PS or IA. Surprisingly, renal allograft survival rates were similar among patients receiving IVIG alone and those receiving the combination of PS, IA, or both followed by IVIG (*p* = 0.18, Figure 3A).

### Adjunctive Immunosuppressive Therapy Did Not Achieve Better Allograft Survival Than Standard ABMR Treatment With PS/IA and IVIG Alone

For 38 of 102 allograft recipients, standard treatment with PS/IA and IVIG was followed by adjunctive immunosuppressive therapy consisting of rituximab (*n* = 10), bortezomib (*n* = 11), thymoglobulin (*n* = 9), or eculizumab (*n* = 3) (Table 1). We found that the application of additional immunosuppressive therapy, such as bortezomib, rituximab, thymoglobulin, or eculizumab, did not achieve better renal allograft survival rates than did standard treatment with PS/IA and IVIG alone (*p* = 0.83, Figure 3B). Moreover, analyses of the subgroups determined by DSA status found no differences in allograft survival rates between patients receiving additional immunosuppressive therapy and those receiving standard therapy (data not shown).

### Increased Maintenance Immunosuppression Exerted a Beneficial Effect on Allograft Survival Among Recipients With DSA-Negative ABMR

**Figure 4:**
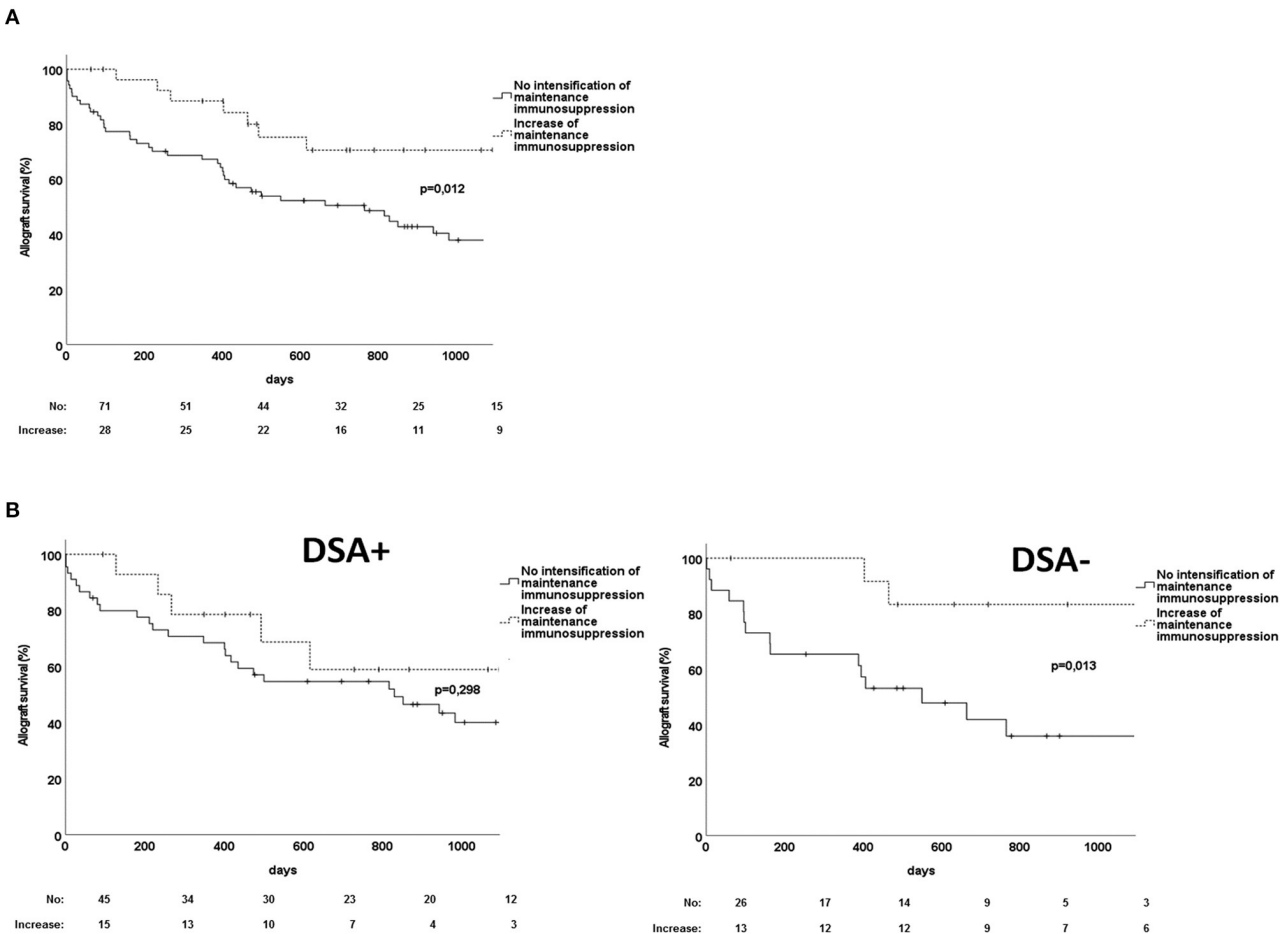
Effect of the increase of maintenance immunosuppression on renal allograft survival among recipients with persistent antibody-mediated rejection. **(A)** Comparison of allograft survival between recipients who were treated with intensified maintenance immunosuppression vs. recipients without increase of maintenance immunosuppression. **(B)** Comparison of the effect of intensified maintenance immunosuppression on allograft survival between recipients with DSA-positive vs. DSA-negative ABMR. ABMR, antibody-mediated rejection; DSA, donor-specific antibody.

Renal allograft recipients exhibiting histologic features of biopsy-proven persisting ABMR received increased maintenance immunosuppression, long-term therapy with IVIG for more than 1 year, or both. Increased immunosuppression was defined as a change in the maintenance immunosuppressive regimen or as a switch from dual to triple immunosuppressive therapy. An acceleration of the dosage of maintenance immunosuppressive drugs was not considered for the analysis. Maintenance immunosuppressive therapy was intensified for 28 patients in the study cohort. These 28 recipients exhibited higher rates of allograft survival than did the remaining 71 patients (*p* = 0.01, Figure 4A). In addition, univariate analysis showed that intensification of maintenance immunosuppression exerted a protective effect on allograft survival among recipients with ABMR (HR, 0.37; *p* = 0.02; Table 3). However, the results could not be confirmed by multivariate Cox regression analysis (Table 3).

With regard to the DSA status of ABMR, we observed significantly better allograft survival after increased maintenance immunosuppression for recipients with DSA-negative ABMR at the 3-year follow-up (*p* = 0.01, Figure 4B). Moreover, both univariate and multivariate analysis detected a positive effect on renal allograft survival when persistent DSA-negative ABMR was treated with increased maintenance immunosuppression (Table 4). In contrast, intensification of maintenance immunosuppression did not influence allograft survival among patients with DSA-positive ABMR (*p* = 0.3, Figure 4B).

**Table 4:** Results of univariate and multivariate analyses identifying risk factors for allograft failure and assessing treatment effects of increased maintenance immunosuppression and long-term therapy with IVIG on allograft survival in the subgroup of 61 recipients with DSA-positive ABMR and 41 recipients with DSA-negative ABMR.

	**DSA**^**+**^ **ABMR**			**DSA** ^**−**^ **ABMR**		
	***n* = 61**			***n* = 41**		
	**HR**	**CI (95%)**	***p*-value**	**HR**	**CI (95%)**	***p*-value**
**Univariate analysis**						
*Risk factors*						
Previous transplants	0.87	0.3–2.9	0.815	1.14	0.4–3.3	0.811
C4d^+^	**3.35**	**1.6–6.9**	**0.001**	1.3	0.5–3.7	0.627
ABMR ≥ 1 year	3.53	0.5–26.1	0.216	**3.57**	**1.2–11.0**	**0.026**
Banff category 2	0.61	0.3–1.3	0.180	0.57	0.2–1.6	0.291
Banff category 2 + 3/4	1.07	0.4–2.6	0.886	1.05	0.4–2.9	0.925
Banff category 2 + 5/6	1.36	0.6–3.3	0.509	1.61	0.5–5.0	0.412
Banff category 2 + 3/4 + 5/6	3.22	1.0–10.8	0.059	1.68	0.4–7.4	0.495
Recipient age at the time of biopsy ≥ 50	1.15	0.6–2.4	0.714	0.78	0.3–2.1	0.622
Acute ABMR	0.87	0.4–1.8	0.699	1.03	0.4–2.7	0.953
Acute+chronic-active ABMR	1.23	0.6–2.7	0.605	1.2	0.4–3.4	0.737
Chronic-active ABMR	0.97	0.5–2.1	0.949	0.7	0.2–3.1	0.632
**Therapy regimen**						
Add-on therapies	1.09	0.5–2.2	0.823	0.67	0.2–1.9	0.452
Intensification of maintenance immunosuppression	0.6	0.2–1.6	0.304	**0.19**	**0.04–0.8**	**0.027**
Long-term IVIG	**0.41**	**0.2–0.9**	**0.021**	1.34	0.5–3.8	0.581
*Multivariate analysis*						
ABMR ≥ 1 year	.	.	.	2.97	1.0–9.2	0.059
Intensification of maintenance immunosuppression	.	.	.	**0.22**	**0.05–1.0**	**0.048**
C4d^+^	**3.37**	**1.6–6.9**	**0.001**	.	.	.
Long-term IVIG	**0.41**	**0.2–0.9**	**0.021**	.	.	.

*ABMR, antibody-mediated rejection; CI, confidence interval; DSA, donor-specific antibody; HR, hazard ratio; IVIG, intravenous immune globulin*.

*Bold values are significant values (p < 0.05)*.

### Long-Term Therapy With IVIG Improved Allograft Survival Among Patients With DSA-Positive ABMR

**Figure 5:**
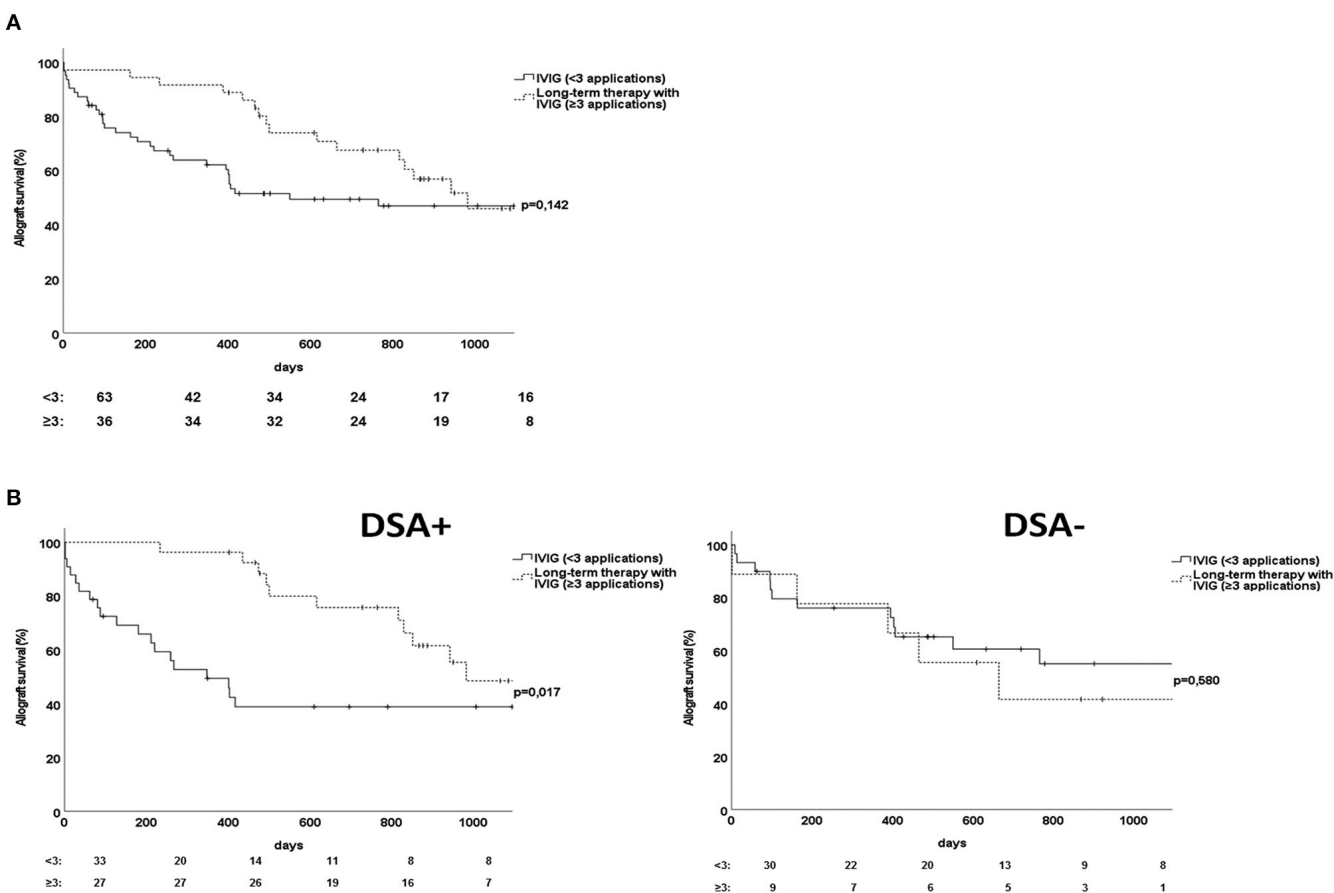
Effect of long-term therapy with IVIG on renal allograft survival of recipients with persistent antibody-mediated rejection. **(A)** Comparison of allograft survival between recipients who were treated with repetitive applications of IVIG over more than 1 year vs. recipients without long-term therapy with IVIG. **(B)** Comparison of the effect of long-term therapy with IVIG on allograft survival between recipients with DSA-positive vs. DSA-negative ABMR. ABMR, antibody-mediated rejection; DSA, donor-specific antibody; IVIG, intravenous immune globulin.

Long-term application of IVIG for more than 1 year was a treatment option for recipients with ABMR as detected by a follow-up biopsy performed after the administration of the standard combination therapy of PS/IA with IVIG. Long-term therapy with IVIG was administered to a total of 34 recipients. However, allograft survival rates did not differ between recipients who received IVIG fewer than 3 times and recipients treated with long-term IVIG therapy (*p* = 0.14, Figure 5A).

After differentiation for DSA status, allograft survival was significantly better among patients with DSA-positive ABMR treated with long-term IVIG than among those treated with short-term IVIG (*p* = 0.02, Figure 5B). Excluding patients with prognostic favorable early ABMR confirmed the advantage of long-term compared to short-term IVIG treatment for late DSA-positive ABMR (*p* = 0.006, Supplementary Figure 1). The protective effect of repetitive administration of IVIG for more than one year as detected by univariate analysis was also detected by multivariate Cox regression analysis for allograft survival (Table 4). Long-term application of IVIG had no effect on allograft survival among patients with DSA-negative ABMR (Figure 5B). It should be mentioned that the subgroup of patients with DSA-positive ABMR contained significantly more recipients who were treated with repetitive IVIG applications than did the subgroup of patients with DSA-negative ABMR (Table 1).

## Discussion

The results of this study showed that a diagnosis of ABMR later than the first year after transplant and C4d positivity as detected by renal allograft biopsy are important indicators of the risk of allograft loss among ABMR patients. Our stepwise treatment analysis showed that the use of adjunctive immunosuppressive therapy, such as rituximab, bortezomib, eculizumab, or thymoglobulin, exerts no additional benefit on graft survival than does treatment with PS/IA and IVIG alone. Our analysis of additional therapeutic effects showed that long-term application of IVIG is more favorable for patients with DSA-positive ABMR, whereas intensification of maintenance immunosuppression therapy is more effective for recipients with DSA-negative ABMR.

We assessed the effect of several ABMR-associated factors on long-term renal allograft survival after the diagnosis of ABMR. Allograft survival rates were similar between DSA-negative ABMR and DSA-positive ABMR. Like us, Crespo et al. and Sablik et al. described a lack of association between DSA status and allograft survival in the presence of ABMR (11, 13). However, our results disagree with those of Senev et al., who suggested that the risk of allograft failure was significant lower for patients with DSA-negative ABMR than for those with DSA-positive ABMR (12). Senev et al. found that DSA-negative ABMR had a transient histologic picture and was less likely to become chronic (12). The discrepancy in the results may be attributed to differences in study design. Senev et al. analyzed protocol biopsies; they included predominantly patients with active ABMR without chronicity and evaluated allograft survival early after transplant (12). In contrast, we included allograft recipients with a biopsy for cause early and late after transplant and followed them for at least 3 years after the detection of ABMR and found that most of them exhibited concomitant histologic features of active and chronic ABMR.

We also found that the timepoint of ABMR diagnosis is a crucial factor determining allograft outcome. Our observation is concordant with those of others showing that the late appearance of ABMR after transplant is a significant risk factor for rapid allograft loss (23, 24).

We found that C4d status as detected by biopsy plays a relevant role in allograft outcome after the occurrence of ABMR. Accordingly, C4d-negative ABMR seems to be an advantageous factor as compared with C4d-positive ABMR. These results are in line with those of a number of previous reports acknowledging C4d-positive ABMR as more severe and as associated with a shortened allograft half-life and microvascular inflammation (25–28).

Furthermore, we found that histologic signs of ABMR in combination with T cell–mediated rejection are not linked with poorer allograft survival. Our results contradict those of Matignon et al., who found that the presence of histologic features of T cell–mediated rejection in addition to C4d-positive ABMR is a risk factor for premature allograft failure (29). These discordant results can be partly explained by our inclusion of C4d-positive and -negative ABMR and the relatively small number of patients with C4d-positive ABMR in our cohort. Additionally, we observed a slight trend toward a higher portion of female recipients in the group of patients with DSA-negative-ABMR compared to patients having ABMR with evidence of DSA. This increase may be related to reactivation of memory B cells to non-HLA antigens to which multiparous females have been previously exposed during pregnancy (16).

In the second step, we determined the effect of various ABMR treatment approaches on allograft survival. As expected, treatment of ABMR with PS/IA in combination with IVIG is superior to no treatment. Although treatment concepts for ABMR vary widely, most centers use a combination of PS and IVIG for treating ABMR. In addition, the Transplantation Society working group recommend PS followed by IVIG as the standard of care for removing circulating DSAs (30). However, this recommendation is based on the results of a few randomized controlled trials showing the effectiveness of PS and IVIG for treating ABMR (31–33). Surprisingly, allograft outcome after treatment with the combination of PS/IA with IVIG was not better than after the administration of IVIG alone. In the early 1990s, the immunomodulatory effects of IVIG on T and B cells were recognized. IVIG can initiate apoptosis of B cells and can modulate B-cell signaling (34). The research groups of Peraldi et al. and Jordan et al. published the first reports showing that treatment with high-dose IVIG led to improvement in renal allograft survival after 5 years' follow-up (35). Cooper et al. and Stegall et al. investigated the effect of high-dose IVIG on the production of DSAs and found a modest decrease in DSAs (36). However, the results of Lefaucheur et al. contradict our results. Treating ABMR with high-dose IVIG alone was inferior to treatment with regimens using a combination of IVIG with PS and rituximab (37). Even so, studies comparing the use of IVIG alone with standard therapy combining PS and IVIG are rare, and the question of whether the administration of IVIG alone is as effective as standard treatment for ABMR should be addressed in future trials.

Several centers have used adjunctive treatment strategies for ABMR, predominantly rituximab in combination with plasmapheresis and IVIG. Our study found that adjunctive therapy strategies for ABMR exerted no beneficial effect on allograft survival. We also noted no beneficial effect of additional treatment with rituximab for the entire cohort or for the subgroups with DSA-positive or DSA-negative ABMR. However, the findings about adjunctive therapy of ABMR with rituximab are controversial. The first controlled trial, performed by Lechaufeur et al., found that survival rates were better when rituximab was added to IVIG and PS (37). Subsequent studies of the effect of additional treatment with rituximab were disappointing. In line with our findings, a phase III, multicenter double-blind study by Sautenet et al. found that rituximab had no favorable effect on allograft survival among patients with ABMR and that serious opportunistic infections occurred more often among patients treated with rituximab (38). Similarly, Wan et al. performed a systematic review evaluating the effect of additional treatment with rituximab and found no significant difference between rituximab-treated recipients and recipients receiving standard of care with IVIG and PS (18). The Spanish multicenter, prospective, double-blind TRITON trial performed by Moreso et al. found that, among patients with chronic ABMR, combined therapy with rituximab did not achieve any improvement in allograft outcome (39). The poor effect of rituximab treatment on ABMR reported in the studies might be attributed the fact that the anti-CD20-antibody is not able to reach plasma cells as the main source of DSA because plasma cells are not expressing CD20 on their surface. Pineiro and colleagues performed a study involving a cohort of 62 patients with chronic active ABMR and found that allograft survival was not significantly affected by rituximab treatment compared to treatment with standard therapy (40). Our study did not differentiate between patients with acute or chronic ABMR, but most of our patients exhibited histologic signs of acute and chronic active ABMR simultaneously.

Besides additional therapy with rituximab, in this study we alternatively used other therapies, including bortezomib, eculizumab, or thymoglobulin. Again, allograft survival rates did not differ significantly between the various treatment strategies for ABMR. With respect to the additional treatment of acute ABMR with bortezomib, several case reports and case series have demonstrated a lower risk of allograft loss after such treatment (41–43). However, in their single-center double-blind BORTEJECT trial, which enrolled 44 renal allograft recipients with late ABMR, Eskandary et al. found similar response rates between a bortezomib-treated group and a group given placebo (44). These recent findings are consistent with our results.

One alternative treatment approach is eculizumab, which targets the complement pathway as a key effector pathway of the ABMR process. A small nonblinded retrospective study by Kulkarni et al. found that eculizumab therapy did not counteract the decrease in eGFR among patients with chronic ABMR associated with *de novo* DSA (45). The ABMR rate in the first 3 months after transplant was significantly lower among patients treated with eculizumab than among historical control subjects treated with PS only (46, 47). However, this effect had disappeared at the follow-up visit 1 to 2 years after transplant. We found that therapy with eculizumab did not significantly affect the allograft survival rates among patients with ABMR, although the results are difficult to interpret because of small patient numbers.

We saw that adjunctive therapy with thymoglobulin failed to achieve a significant improvement in allograft survival rates among some recipients with ABMR. Cihan et al. found a significant amelioration of allograft function in 4 of 9 pediatric kidney transplant recipients with chronic ABMR, but larger studies are lacking (48).

One of the main findings of our analysis of secondary therapies for persistent ABMR was the positive effect of increased maintenance immunosuppression on allograft survival among recipients with ABMR. In most cases, double immunosuppressive therapy consisting of a calcineurin inhibitor and a steroid was supplemented by MMF/MPA. Supporting our data is the finding that immunosuppression with MMF is associated with a decrease in allograft failure that can be partly attributed to the reduction of rejection rates among recipients treated with MMF (49). Moreover, as had been shown by Briggs et al., switching immunosuppressive therapy from cyclosporine to tacrolimus reduces the risk of acute rejection (50). The use of a combination of sirolimus and tacrolimus is known to exert a weaker immunosuppressive effect than that of tacrolimus combined with MMF and to contribute to poorer allograft survival (51). The Transplantation Society working group also came to the consensus that optimization of baseline immunosuppressive therapy is indicated for patients with chronic active ABMR (1).

In particular, the subgroup of recipients in whom DSA-negative ABMR developed benefited from intensification of maintenance immunosuppression. We can speculate that the observed positive effect on allograft outcome is linked to the immunomodulatory effects of intensified maintenance immunosuppression in reducing unidentified triggers of DSA-negative ABMR.

In DSA-positive ABMR, long-term treatment with IVIG exhibited a sustained positive treatment effect compared to short-term IVIG treatment. Therapy with IVIG has been shown to suppress the production of anti-HLA antibodies and to stop the evolution of acute and chronic ABMR (52). Nevertheless, no clinical studies to date have shown that the repetitive administration of IVIG has an advantageous effect on allograft survival after the occurrence of ABMR. A retrospective analysis by Sablik et al. (53) found that allograft function recovered after the administration of IVIG to kidney transplant recipients with ABMR. Long-term therapy with IVIG may induce immunomodulatory effects on DSA production and slow the progression of ABMR (36). However, these results should be interpreted with caution, because significantly more patients with DSA-positive ABMR were treated with IVIG repetitively as a secondary therapy for ABMR. This potential bias may have influenced our observations. Additional clinical trials are necessary to clarify the effect of long-term IVIG therapy on allograft survival and allograft function among patients with ABMR.

We are aware that this study has several limitations. The main limitation is the retrospective study design, which complicates comparisons of our results with those of previous prospective double-blind clinical trials investigating the effect of various therapeutic approaches for ABMR on renal allograft survival. Another limitation is the fact that DSA-negative patients were significantly older than DSA-positive patients, and the subgroup analyses had to take this fact into account. In addition, a significantly higher portion of recipients with DSA-negative ABMR received a second transplant, and early ABMR was diagnosed more frequently in this subgroup. Long-term therapy with IVIG was administered significantly more frequently to DSA-positive recipients than to DSA-negative recipients.

Thus, our study is characterized by potential selection bias because of the retrospective study design that may have affected the differences between the two subgroups in their responses to intensification of immunosuppression and long-term application of IVIG. It should also be noted that only indication biopsies were performed at our center. Otherwise, in assessing the effectiveness of various primary and secondary therapies for ABMR we did not discriminate between early and late ABMR or between the histologic phenotypes of ABMR according to the Banff classification. We focused on the relevance of DSA status to the differences in therapeutic responsiveness of ABMR. Several recipients exhibited morphologic signs of acute ABMR in parallel with chronic-active ABMR or ABMR mixed with T cell–mediated rejection. Of note, 46% of all transplant recipients with DSA-negative ABMR exhibited early ABMR within the first year after transplant; this selection bias regarding a positive ABMR outcome may have skewed these results. Our study did not evaluate new promising treatment options for ABMR, such as tocilizumab, a humanized monoclonal antibody targeting the interleukin-6 receptor (54, 55).

To summarize, our retrospective cohort study showed that the prognosis for patients with ABMR is poor regardless of DSA status; it also confirmed that prognostic factors of ABMR, such as timepoint of diagnosis and C4d status, are clinically relevant. Interestingly, we observed that long-term administration of IVIG to recipients of DSA-positive allografts with preferentially late ABMR exerts a positive effect, whereas intensification of maintenance immunosuppression therapy is more effective for recipients with DSA-negative ABMR. Additional studies of long-term treatment for ABMR are needed and should consider DSA status in addition to histologic signs of ABMR.

## Data Availability Statement

The original contributions presented in the study are included in the article/Supplementary Material, further inquiries can be directed to the corresponding author/s.

## Ethics Statement

The studies involving human participants were reviewed and approved by Institutional Ethics Board of the University Hospital Duisburg-Essen, Germany. Written informed consent for participation was not required for this study in accordance with the national legislation and the institutional requirements.

## Author Contributions

MK, JF-K, and UE contributed to conception and design of the study, performed the statistical analysis, and wrote the first draft of the manuscript. MK organized the database. JHB was responsible for banff grading of renal pathology. All authors contributed to manuscript revision, read, and approved the submitted version.

## Funding

JF-K was supported by the Clinician Scientist Program of the University Medicine Essen Clinician Scientist Academy (UMEA).

## Conflict of Interest

The authors declare that the research was conducted in the absence of any commercial or financial relationships that could be construed as a potential conflict of interest.

## Publisher's Note

All claims expressed in this article are solely those of the authors and do not necessarily represent those of their affiliated organizations, or those of the publisher, the editors and the reviewers. Any product that may be evaluated in this article, or claim that may be made by its manufacturer, is not guaranteed or endorsed by the publisher.

## Supplementary Material

Supplementary Figure 1 Comparison of allograft survival between recipients who were treated with repetitive applications of IVIG over more than 1 year vs. recipients without long-term therapy with IVIG in the subgroup of patients having late ABMR and positive DSA status.

## Abbreviations

ABMR: antibody-mediated rejection
CI: confidence interval
DSA: donor-specific antibody
CNI: calcineurin-inhibitor
HLA: human leukocyte antigen
HR: hazard ratio
IA: immunoadsorption
IgG: immunoglobulin
IQR: interquartile range
IVIG: intravenous immunoglobulin
MAC: membrane attack complex
MFI: mean fluorescence intensity
MMF: mycophenolate mofetil
MPA: mycophenolic acid
PRA: panel-reactive antibodies
PS: plasmapheresis
SAB: single-antigen bead
Tx: transplantation.
