# Supplementary figures and images for: Differential Treatment Effects for Renal Transplant Recipients With DSA-Positive or DSA-Negative Antibody-Mediated Rejection
Source: Front Med (Lausanne). 2022 Jan 31;9:816555. doi: 10.3389/fmed.2022.816555 (PMC8841765; doi:10.3389/fmed.2022.816555)

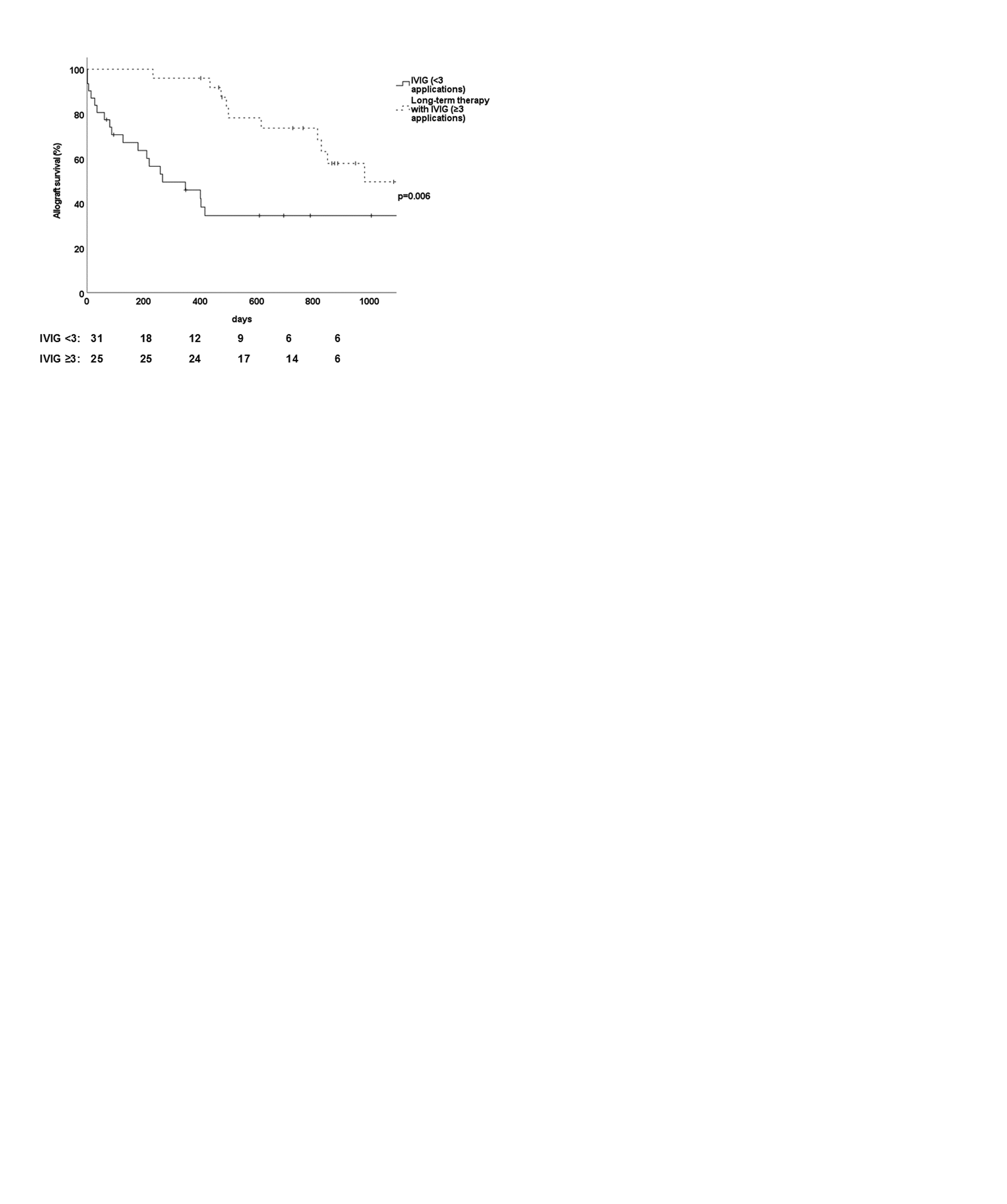

Supplement: Supplementary Figure 1 — Comparison of allograft survival between recipients who were treated with repetitive applications of IVIG over more than 1 year vs. recipients without long-term therapy with IVIG in the subgroup of patients having late ABMR and positive DSA status. [file Image_1.TIF]
